# The Russia-Ukraine Conflict: Its Implications for the Global Food Supply Chains

**DOI:** 10.3390/foods11142098

**Published:** 2022-07-14

**Authors:** Sandeep Jagtap, Hana Trollman, Frank Trollman, Guillermo Garcia-Garcia, Carlos Parra-López, Linh Duong, Wayne Martindale, Paulo E. S. Munekata, Jose M. Lorenzo, Ammar Hdaifeh, Abdo Hassoun, Konstantinos Salonitis, Mohamed Afy-Shararah

**Affiliations:** 1 Sustainable Manufacturing Systems Centre, School of Aerospace, Transport & Manufacturing, Cranfield University, Cranfield MK43 0AL, UK; k.salonitis@cranfield.ac.uk (K.S.); m.a.shararah@cranfield.ac.uk (M.A.-S.); 2 Department of Work, Employment, Management and Organisations, School of Business, The University of Leicester, University Road, Leicester LE1 7RH, UK; ht203@leicester.ac.uk; 3 Glenfield Hospital, University Hospitals of Leicester, NHS Trust, Leicester LE3 9QP, UK; ftrollman@gmail.com; 4 Department of Agrifood System Economics, Centre ‘Camino de Purchil’, Institute of Agricultural and Fisheries Research and Training (IFAPA), 18080 Granada, Spain; guillermo.garcia@juntadeandalucia.es (G.G.-G.); carlos.parra@juntadeandalucia.es (C.P.-L.); 5 Faculty of Business and Law, The University of the West of England, Bristol BS16 1QY, UK; linh.duong@uwe.ac.uk; 6 National Centre for Food Manufacturing, University of Lincoln, Holbeach PE12 7PT, UK; wmartindale@lincoln.ac.uk; 7 Centro Tecnológico de la Carne de Galicia, Rúa Galicia n 4, Parque Tecnológico de Galicia, San Cibrao das Viñas, 32900 Ourense, Spain; paulosichetti@ceteca.net (P.E.S.M.); jmlorenzo@ceteca.net (J.M.L.); 8 Area de Tecnoloxía dos Alimentos, Facultade de Ciencias, Universidade de Vigo, 32004 Ourense, Spain; 9 Agri-Food Sustainability Assessment, University de Lorraine, 54600 Nancy, France; a.hdaifeh@saf-ir.com; 10 Sustainable AgriFoodTech Innovation & Research (SAFIR), 62000 Arras, France; a.hassoun@saf-ir.com; 11 Syrian Academic Expertise (SAE), Gaziantep 27200, Turkey

**Keywords:** conflict, consumer, food processing, food production, food logistics, food quality, food storage, food supply chain, Russia, Ukraine, war

## Abstract

Food is one of the most traded goods, and the conflict in Ukraine, one of the European breadbaskets, has triggered a significant additional disruption in the global food supply chains after the COVID-19 impact. The disruption to food output, supply chains, availability, and affordability could have a long-standing impact. As a result, the availability and supply of a wide range of food raw materials and finished food products are under threat, and global markets have seen recent increases in food prices. Furthermore, the Russian-Ukrainian conflict has adversely affected food supply chains, with significant effects on production, sourcing, manufacturing, processing, logistics, and significant shifts in demand between nations reliant on imports from Ukraine. This paper aims to analyze the impacts of the conflict between Russia and Ukraine on the effectiveness and responsiveness of the global food supply chains. A PRISMA (Preferred Reporting Items for Systematic Reviews and Meta-Analyses) approach, including grey literature, was deployed to investigate six key areas of the food supply chains that would be impacted most due to the ongoing war. Findings include solutions and strategies to mitigate supply chain impacts such as alternative food raw materials, suppliers and supply chain partners supported by technological innovations to ensure food safety and quality in warlike situations.

## 1. Introduction

The current Russian invasion of Ukraine that began on 24 February 2022 has led to the loss of lives, property, assets and infrastructure in Ukraine, resulting in a humanitarian disaster. It is rapidly becoming the most severe refugee crisis in Europe since World War 2 [1]. It has disrupted agriculture (both countries are globally important producers of agricultural commodities), and this has impacted the food supply chain, particularly those nations dependent on sunflower oil, maize, and wheat. The current sanctions and banning of trading of Russian and Belarussian (an ally of Russia) agricultural goods via the USA, European Union and allies of it has created a shortfall on various food commodities such as wheat and cooking oil [2]. Increases in food and fuel prices have also occurred, Russia is a primary supplier of gas to Europe, and the combined impact of conflict and sanctions has seen 3–4-fold increases in energy prices [3]. The most exposed are countries that heavily depend on agricultural exports from Ukraine and Russia for their food and feed industries, as well as those that are dependent on nitrogenous fertilizers from Russia and Belarus for agricultural production [4].

Previous food supply chain shocks and price increases are well characterized and include droughts, floods and conflict in 2007 and 2008 across several nations resulting in the banning of food export and subsequent increase in fuel prices and rapid two-fold increases in food prices [5]. COVID-19 onset in 2020 added to the economic and agricultural supply chain shocks [6]. The Russia-Ukraine war is compounding these issues and endangering food security worldwide by the acute increases in food prices for the most vulnerable globally. It is impacting political stability worldwide, such as ousting of Pakistan’s Prime Minister Imran Khan, unrest in Peru and Sri Lanka all have the implication of increased high food and fuel prices being drivers of unrest. It has led to a flashpoint in France, Germany, Italy and Spain, where resulting energy allowances, decreases in prices, and taxes amount to fiscal intervention by governments to address increasing energy prices [7]. Consequently, the scale and potential scope of impacts are without recent precedent, and a research gap exists in that a comprehensive investigation of the effects of such disruption needs to be strengthened in the current literature.

In this context, this paper addresses the impacts of the Russia-Ukraine conflict on all the actors of the food supply chain and will discuss solutions and strategies to counter these impacts. The hypothesis underlying this research is that there will be significant impacts on the effectiveness and responsiveness of global food supply chains as a result of the conflict between Russia and Ukraine. The related research question is as follows. What strategies or solutions may be implemented to mitigate the impacts of the conflict between Russia and Ukraine on global food supply chains?

## 2. Methodology

Large-scale review syntheses, especially those concerning timely and evolving topics, have grey literature as an important source of information [8]. A grey literature search plan should outline the resources, search terms, websites, and limits to be used [9]. Elements to be reported according to a PRISMA (Preferred Reporting Items for Systematic Reviews and Meta-Analyses) include a description of all information sources in the search, the name of the person conducting the search, the date the search was performed, and a full search strategy of at least one database including all search terms and combinations [10].

The impact categories that are the focus of this research were initially identified with expert consultation using a qualitative two-round e-Delphi method [11,12] with 13 participants. The first round was used to identify potential impacts of the Russia-Ukraine conflict on food supply chains based on the hypothesis that significant impacts on the effectiveness and responsiveness of global food supply chains are the result of the conflict between Russia and Ukraine. The second round asked participants to rank the impacts identified in the first round. The top six impacts defined the impact areas followed up in the review synthesis.

A grey literature search plan was developed incorporating three different searching strategies: (1) customized Google search, (2) targeted websites, and (3) news reports. The grey literature search was supplemented with relevant peer-reviewed articles from the SCOPUS and Google Scholar databases using the same search terms: “Ukrain*”, “Russi*”, “war”, and the keywords of each of the identified impact areas. Each impact area was searched in this way by one of the co-authors based on their area of expertise, supported and corroborated by the other co-authors. Searches were performed from 13 March 2022 to 14 May 2022.

This research employed a PRISMA approach with the eligibility criteria shown in Table 1.

## 3. Results

### 3.1. Results of the e-Delphi

**Figure 1:**
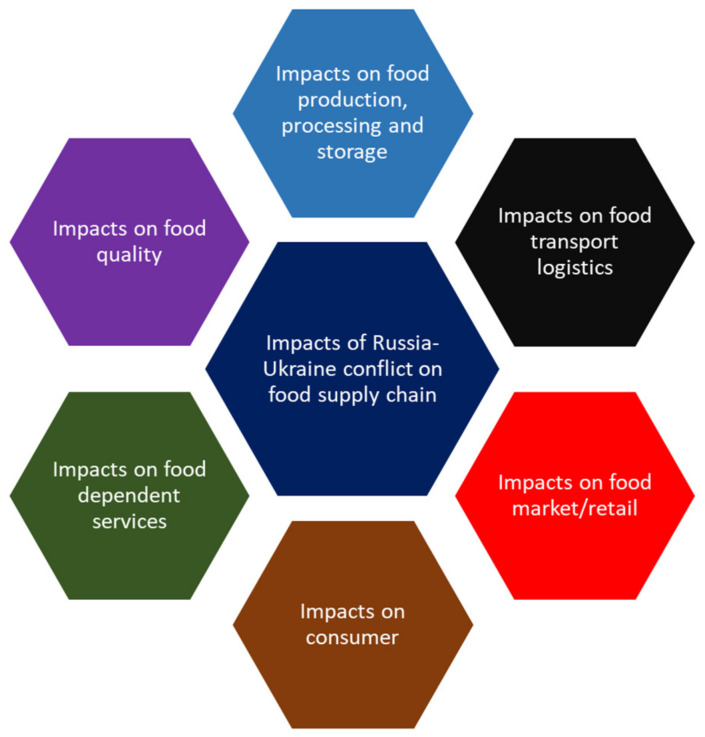
Impacts of Russia-Ukraine conflict on the food supply chain.

The participants in the e-Delphi identified the top six supply chain impacts of the Russian invasion of Ukraine, as shown in Figure 1.

Additional impacts that were not part of the top six impacts included impacts on sustainability.

### 3.2. Results of the Review Synthesis

The following sections present the results of the review synthesis (literature review) for each of the six impacts shown in Figure 1.

#### 3.2.1. Impact on Food Production, Processing, and Storage

Ukraine has a major role in Europe and beyond in terms of agriculture and food production. The national capability to cultivate soils, sow, and harvest crops has been reduced since the start of the war [13]. Major food security concerns are the disruption to winter harvesting and spring planting, availability of agricultural labor, availability of agricultural inputs, damage to crops due to military activity, and destruction of food system assets and infrastructure [13]. A consequence of these outcomes is food shortages for the local population and export economy, both in the short and medium-term.

Around 70% of Ukraine’s land is agricultural, which is similar to other European nations such as the UK, this includes cultivated land (grains, technical crops, forages, potatoes and vegetables, and fallow), gardens, orchards, vineyards, meadows, and pastures [14]. The agricultural and food sector plays a very important role in Ukraine’s economy. Agriculture, forestry, and fishing was Ukraine’s third-largest sector by gross value added in 2020, which differentiates it from nations such as the UK where direct agriculture represents less than 5% of the GDP [15]. In Ukraine, agriculture contributed to the gross domestic product with around 9.3% and employed nearly 17% of the working population [16]. Ukraine exported more than $9.4bn worth of cereals in 2020, around one-fifth of its total exports, and it is ranked second global cereal exporter, whereas the US is first ranked [15]. The impacts of the war in Ukraine’s agricultural and food sectors are also expected to affect the economy of the country significantly.

In the short term, the main food-security concern in Ukraine is food access, not food availability, since available stocks in Ukraine were approximately 1.14-fold greater than the estimated annual demand [13]. Restriction of labor and reduced availability of workers, due to population fighting in the war and concerns over the safety of the workers that stayed on farms, is a critical reason why crops are not harvested. To partially alleviate this problem, the Ukrainian government gave an exemption to farmers, so they do not have to join the armed forces [17]

The main small-grain crops planted in Ukraine are winter wheat, spring barley, and maize [14]. Although wheat is grown throughout the country, production in central and south-central areas of the country dominates. There are currently six million hectares of wheat planted in Ukraine [18]. Barley has been the most consumed feed grain in Ukraine for most of the past ten years [14]. Spring barley is planted mostly in eastern Ukraine, in some of the most-affected war areas.

FAO [4] estimates that 20–30% of the areas allocated to winter cereal, maize, and sunflower seed production will not be harvested or planted this spring. Furthermore, a lack of fertilizers and pesticides will affect the crops’ yield. The availability of seeds for planting in 2022 is relatively high, particularly for vegetable crops, due to available stocks and insignificant import requirements. The main constraint in this aspect is logistics and a shortage of maize and sunflower seeds is expected [13].

The major concerns and impacts caused by the war for the most critical food sectors in Ukraine are shown in Table 2. It demonstrates the impacts have already been felt in most food sectors, and they will continue affecting food production in Ukraine even if the war were to stop.

In addition to agrifood-security concerns in Ukrainian and Russian agrifood sectors, the war has already affected global food supply chains. The major role that Ukraine and Russia play in global food supply chains can be understood using reported production and domestic supply statistics [15,18,19,20]. Ukraine’s wheat production is 3.2% of global production and exports are 9.1% of the global wheat exports [21]. Russia is the first ranked global exporter of wheat, and Ukraine is the fifth ranked exporter and most critically a key supplier of wheat to the World Food Programme. This will intensify global food security pressure and there are currently concerns being aired from the UN with respect to wheat supplies to Syria, which is not alone as the following demonstrates Ukraine’s key position globally.

- Ukraine maize production is 2.6% of global production exporting Ukraine exports around 15% of the global maize and is 5th ranked behind USA, Argentina, and Brazil;
- Ukraine barley production is 4.9% of global production and it is the 2nd ranked global exporter behind France with 13.2% of the total world exports;
- Ukraine produces 29.1% of the global sunflower oil, and exports 44% of the global supply of sunflower oil. Ukraine is the largest exporter of sunflower seeds globally.

Supply is specific and nations directly dependent on these agrifood supplies, including some of the most vulnerable food security countries. For instance, India, China, and some Middle East countries rely on imports of sunflower oil from Russia and Ukraine, while Egypt and Libya obtain two-thirds of their wheat supply from these countries [19]. Lebanon is supplied with 80% of its wheat from Ukraine [18]. Ethiopia and Yemen are vulnerable countries that heavily rely on wheat imports from Ukraine and Russia [22]. Lithuania, China, and India obtain more than 80% of their maize imports from Ukraine. Interestingly, Russia obtains 73% of its barley imports from Ukraine [19].

An increased production in other countries could compensate for this reduction in the availability of foods produced in Ukraine and Russia. The EU has allowed planting and harvesting in fallow land where there was a requirement to leave 5% of the land in an enterprise uncultivated for crops to reduce these current pressures [23,24].

#### 3.2.2. Impact on Food Transport Logistics

The war in Ukraine has created an enormous food security challenge in the country, with important consequences for global food supply chains. The food market has been disrupted by restrictions in export licensing and port closures [13]. Major food security concerns due to the war include disruption of logistics and food supply chains, access to agricultural inputs, abandonment of and reduced access to agricultural land, and destruction of food system assets and infrastructure [13].

The resilience of the Ukrainian domestic food supply can be forecasted because of existing statistical reporting systems associated with a well-developed agri-production industry and its established dependencies across the global food system. There is currently uncertainty regarding the continued scaling up of conflict and its duration but what is known is the Ukrainian agri-production industry depends heavily on fuel imports, with about 70% of imports of petrol and diesel coming from the Russian Federation and Belarus [25]. Fuel supply is critical to any possible spring planting in 2022 and future harvesting in August 2022. This is critical to the Ukrainian Government, which has reduced excise duty and taxes on fuel. FAO reports that only 20% of 1300 large agri-businesses surveyed by the Ukrainian Government have enough fuel for planting this spring [26]. The supply of equipment, seed, plant nutrients, and crop protection supplies are expected to be at least half of that required for full typical planting operations. Whereas smallholder sectors such as potatoes and vegetables will be self-sufficient for seed the internal issue of logistics, being able to transport seed stocks to where they are required will stifle the potential to reach the planting operations. The logistical dependency on fuel will limit the whole agri-food industry and reduced gas supplies will compound this situation by limiting the production of ammonia and urea for nitrogenous fertilizer. This is not just a production issue; it is one of being able to transport commodities and goods to where they need to be used. The war has not only limited the supply of fuel and gas but also destroyed efficient transport infrastructure to farms and factories.

An important current indicator of current production stress is the monthly summary of the Vegetation Condition Index (VCI) at 1 km^2^ resolution [27]. FAO has developed a Country-level Agricultural Stress Index System (ASIS) [28] using satellite data to detect cropped land that could be affected by drought and used to support the Global Information and Early Warning System on Food and Agriculture (GIEWS). The VCI evaluates the current vegetation health compared to the historical trends using the current dekadal Normalized Difference Vegetation Index (NDVI) to its long-term minimum and maximum, so that the data is normalized by the historical range of NDVI values for the same time period. The VCI was designed to separate the weather-related component of the NDVI from the seasonal variation in vegetation cover. The Web Map Service (WMS) was used in this study and analyzed using MapInfo 10.0 [29].

**Figure 2:**
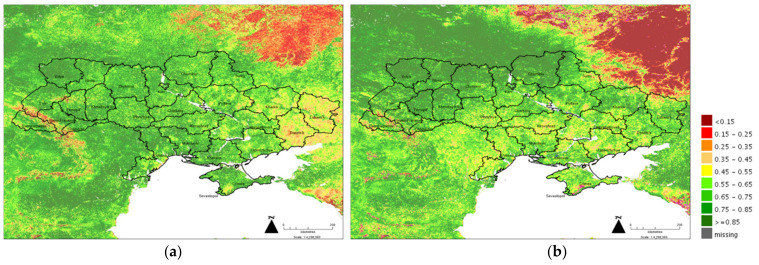
The VCI for March 2021 (**a**) and March 2022 (**b**). The scale for the VCI is shown to demonstrate differences between VCI for each planting season in the Ukrainian Oblasts (regions). Data from UNFAO, analysis developed using MapInfo 10.0.

**Figure 3:**
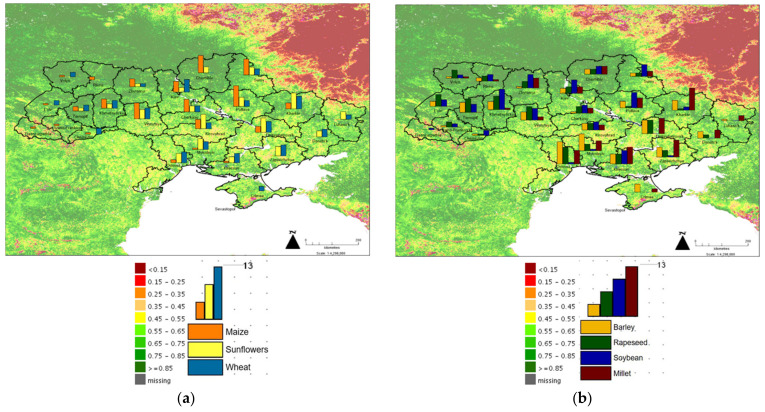
The March 2022 VCI and proportion of maize, sunflowers, and wheat produced in each Oblast (region) (**a**) and March 2022 VCI and proportion of barley, rapeseed, soybean, and millet produced in each Oblast (**b**). The maximum proportion of crop produced is 13% of the total and each bar is relative to this 13% maximum. Data from UNFAO and USDA FAS, analysis developed using MapInfo 10.0.

Crop production data were obtained from the USDA Foreign Agricultural Service [30] which reports the 5-year average (2016–2020) proportion of specific crops produced across the Ukrainian Oblasts (regions) using the State Statistic Service for Ukraine and Rosstat for Crimea Oblast. Figure 2 shows the VCI data for March 2021 (Figure 2a) and March 2022 (Figure 2b). The central Oblasts are where Ukraine’s major agricultural crops are produced and it can be seen that these central regions have a 20–25% reduction in VCI currently suggesting growth and planting is significantly reduced. Figure 3 shows the relative proportions for production of Ukraine’s major crops as presented by the USDA FAS, these include maize, wheat, and sunflowers (Figure 3a) and barley, rapeseed, soybean, and millet (Figure 3b). The VCI grid is shown, demonstrating the Oblasts with the greatest production are regions where there is a reduction in planting and VCI. The key crops impacted provide specific issues that must be resolved but the universal issue is a lack of fuel and ability to transport goods from where they are produced to where they are required [31].

Wheat is typically highlighted by current commenters. Ukraine’s wheat production is 3.3% of the total global wheat production (24.9 × 10^6^ tonnes of global 760,926 × 10^6^ tonnes global crop as reported by FAOStat in 2020, and as the final breaking of wheat embargoes between the USSR and USA in the late 1980s demonstrated. Ukraine is ranked 8th in global wheat production with some 5.5-fold less production than China which produces the greatest amount (134 × 10^6^ tonnes). Logistics and location stifle supply with the UN FAO estimating that 49 percent of winter wheat and 38 percent of rye to be harvested in July–August 2022 are in occupied or war-affected areas (Figure 3a). Most important to the global food system is where this wheat and small grain production is exported, Ukraine is the 5th ranked global exporter of wheat at 18.06 × 10^6^ tonnes in 2020 which is 9% of global wheat exports (FAOStat). Importing nations have a high dependency on this supply that will be limited by potential to harvest and transport. The small grains global market demonstrates how complex geopolitical controls of food and feed supply are with significant volumes of maize from Ukraine to China, Western Europe, and Northern Africa. Most notably the war will restrict shipping from the Black Sea ports. Figure 3a shows the importance of sunflowers in the central and Eastern Oblasts destroyed by war. Sunflowers are typically planted in April and harvested from mid-September to mid-October. This means sunflower production will not be possible. Ukraine supplies 26% of global sunflower seed, exporting seed for processing and oil to India, China, and Western Europe.

#### 3.2.3. Impact on the Food Supply Markets

**Figure 4:**
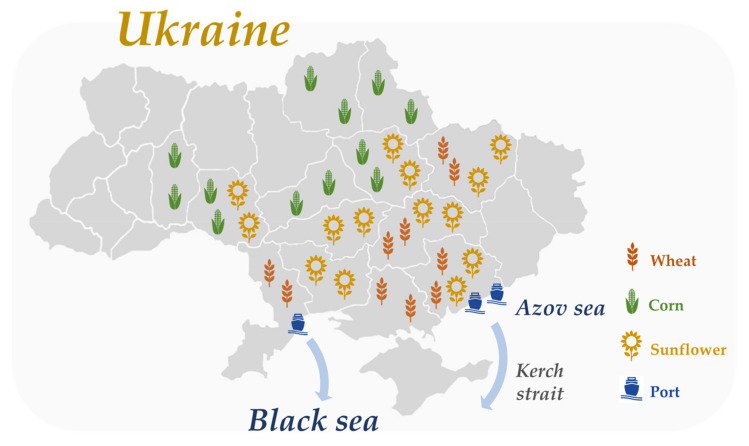
Main regional production areas of wheat, maize, and sunflower, ports at Azov and Black Seas, and maritime routes for Ukrainian agricultural production. Information adapted from International Grains Council [1,2].

The countries involved in the Ukrainian crisis are important global producers of agricultural products [32,33]. Ukraine is a major global producer of wheat, sunflower, and maize that have been mainly transported by ship to ports located at Mariupol and Berdyansk (Azov Sea) and Odessa (Black Sea) (Figure 4).

The European Union (EU) has important commercial relationships with Ukraine, importing wheat, maize, sunflower seed, and oil as well as malt, rye, and sorghum [34,35]. The commercial relationships for the import of commodities between the EU and Ukraine are delivered through duty-free quotas, which provide favorable import outcomes for Ukraine and the EU. The EU has a dependency on commodities and raw materials in the food processing sector [36]. The current uncertain situation about the progression of the armed conflict poses a threat to disrupting agricultural supply chains between Ukraine and European countries [37]. This threat has been gaining momentum since the Russian annexation of the Crimea region in 2014 and which restricted port operations in Mariupol and Berdyansk ports. Consequently, the exports were diverted to Odessa port. The Odessa port has been the most relevant port for Ukrainian exports to Europe since the events in Crimea in 2014 [38].

During the conflict in March 2022, the city and port of Odessa have currently suffered less disruption than other cities in the west of Ukraine. The strategic importance of ports (especially Odessa) would restrict the transport of wheat, cereals, and other commodities trade [38,39]. Currently, the Ukrainian port operations are suspended, and efforts have been made to improve the transport using railways, but the shipping by sea for Ukrainian commodities is limited [33]. The main destinations of Ukrainian wheat, maize, sunflower seeds, and oil in 2020 within the EU were Bulgaria, Cyprus, Estonia, Greece, Hungary, Italy, Lithuania, Netherlands, Portugal, and Spain where Ukraine is also the primary ranked nation for the source of these imported products (Table 3) [21]. Table 3 also demonstrates where there are likely to be indirect pressures on commodity and food markets as the relationships between the EU and Ukraine accommodate the pressure of conflict by sourcing products from different exporting partners [21].

**Figure 5:**
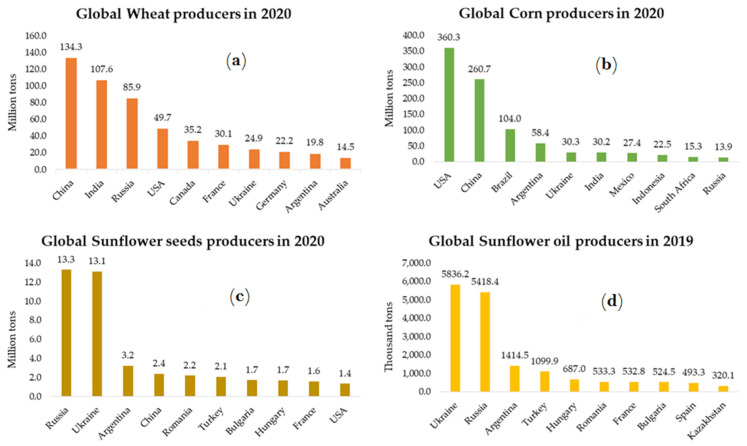
Global producers of wheat (**a**), maize (**b**), sunflower seed (**c**) in 2020, and sunflower oil (**d**) in 2019. Data adapted from FAOSTAT [21].

The international market demonstrates the importance of commodities exported from Ukraine. With the reduced supply of these commodities and continuous demand for them, buyers in European countries are looking for alternative suppliers. Important global producers of these commodities are Argentina, Australia, Brazil, Canada, China, India, Mexico, and the USA (Figure 5).

**Figure 6:**
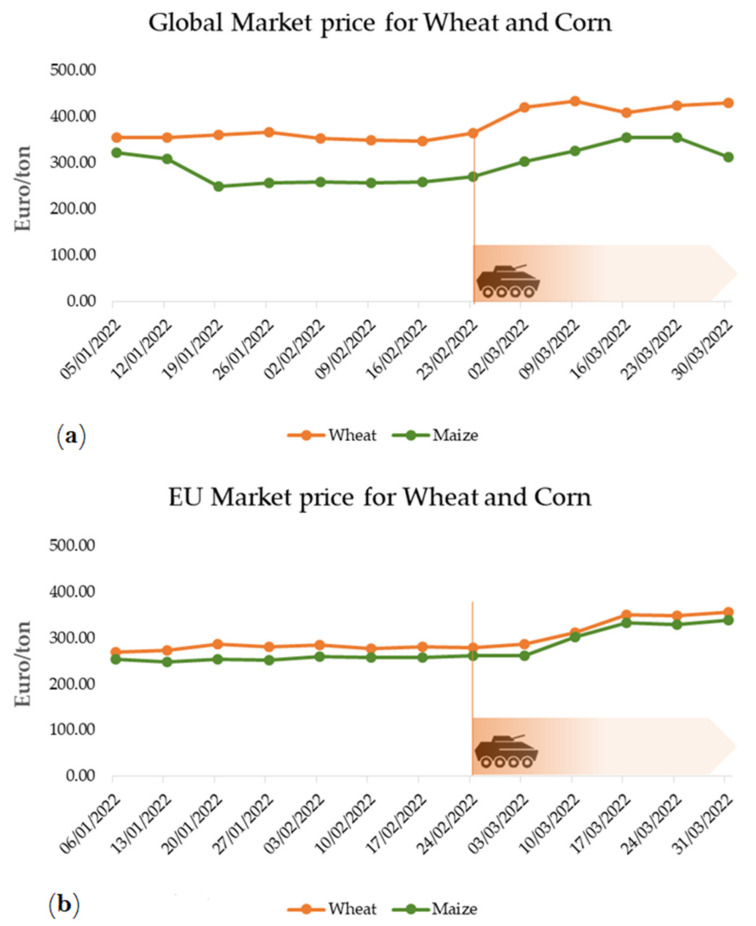
Average global (**a**) and European (**b**) prices for commercialization of wheat and maize during January-March 2022. Data adapted from the Cereal statistic webpage of the European Commission [40].

It is important to consider that European countries and the main global producers of these commodities were not accommodating the absence of Ukrainian commodities for trade offs to be in place and active [35]. Consequently, the market has reacted to the potential reduced supply of Ukrainian commodities for the European countries rather than developing new markets. Commercialization of wheat and maize are affected and general increase in their prices has gained new higher trading levels in relation to values commercialized since the beginning of 2022 [40]. Although differences exist among wheat and maize markets, the effect on prices is impactful Figure 6. As Ukraine’s wheat exportation remain stagnated, the export from major global producers (Australia, India, and Canada) have increased [37].

Within the EU, the supply chains that are partly controlled and determined by Ukrainian agricultural imports include the secondary food processing sector (especially baking, brewing, and vegetable oil industries) which have direct impacts on retail and food service sectors [36]. As the Ukrainian crisis continues, the risk of retail disruption in these food chains increases and leads to a potential downstream shortage of products for industries, grocery and retail stores, and the food service sector.

The threat of supply chain disruption, and the resilience of the European supply chains of wheat, maize and sunflower seed and oil must be considered. Differences in their imports from Ukraine and other European countries (Table 3), most countries in Europe are expected to face future challenges. The lack of supplier diversity is not favorable for the resilience of wheat, maize, and sunflower seed and oil. As sanctions against Russian fuels (coal, oil, and gas) continue, the costs associated with road freight will play an important role in the price of wheat, maize, and sunflower seed and oil to consumers as well as products and food services related to these commodities [41]. Moreover, the processing sector may be affected by strikes of carrier companies (e.g., [42]), which may affect the capacity of food supply chains to thrive in this scenario.

#### 3.2.4. Impact on Consumer

Famine and pestilence have at times been reported as companions to war and death, so it is perhaps unsurprising that the invasion of Ukraine was accompanied by turmoil in markets for food and medicine. Since the war began, wheat prices have risen over forty percent, to prices not seen since the 2007–2008 World Food Price Crisis [43]. Other recent wars such as the Tigray War in Ethiopia or the Nagorno-Karabakh War in Azerbaijan have not been anticipated by market actors to be as disruptive to food and medical supply chains and have been functionally invisible when looking at global markets. However, the Russian invasion of Ukraine is widely predicted to have substantial impacts on global health and food security, and indeed already has.

Before the invasion, Ukraine was the fourth largest exporter of cereals in the world and one of the world’s largest producers, harvesting over 3.7% of the world’s total cereals [44]. The share of the market’s cereal exports is even larger, with 6% of global market food calories coming from Ukraine [45]. Recent events shed light on what massive devastation by the Russian military could mean for Ukraine and the world, including the Syrian Civil War and the 2007–2008 World Food Price Crisis. Syria’s gross exports amounted to $11.9 billion the year before the war, and most recently were measured at $0.6 billion [46]: a real and sustained reduction of over 90% of the country’s exports in the wake of the Russian bombing, which is a plausible outcome for Ukraine as well. Ukraine increased tariffs in 2007 in order to reduce the amount of food exported, and the amount of Ukrainian wheat on international markets was reduced by over $300 million that year as a result. The removal of Ukrainian wheat from export markets was a minor exacerbating factor in the food price crisis, but in the years since the world has become more dependent on Ukrainian cereals. Ukraine exported $761 million worth of wheat in 2006; by 2019 this had grown to $3.1 billion. Further, maize production for export has grown even faster in terms of tonnage and economic value and as of 2019 maize was Ukraine’s largest cereal export at $4.77 billion [47].

While cereal prices have already reacted to the war in Ukraine, these price hikes are to be understood as anticipatory. Ukraine’s largest crop in tonnage and dollar value is maize, which was mostly intended for export and mostly intended as animal feed. Year 2021 already saw maize harvested at some 40 million metric tonnes (MMT), and the next harvest was intended to be in the autumn of 2022. This maize would have been destined to feed animals over the winter, primarily in China (8.4 MMT), Holland (2.3 MMT), Egypt (2.2 MMT), Spain (1.9 MMT), and Iran (1.2 MMT). The shortfall in maize production will thus have direct effects on consumers in meat availability starting in 2023. There is substantial time for would-be importers to arrange alternative sources via global markets, and they will probably do so. For example, three separate American states (Iowa, Illinois, and Nebraska) individually produce more than 40 MMT of maize per year, and the global impact will primarily be financial [48].

**Figure 7:**
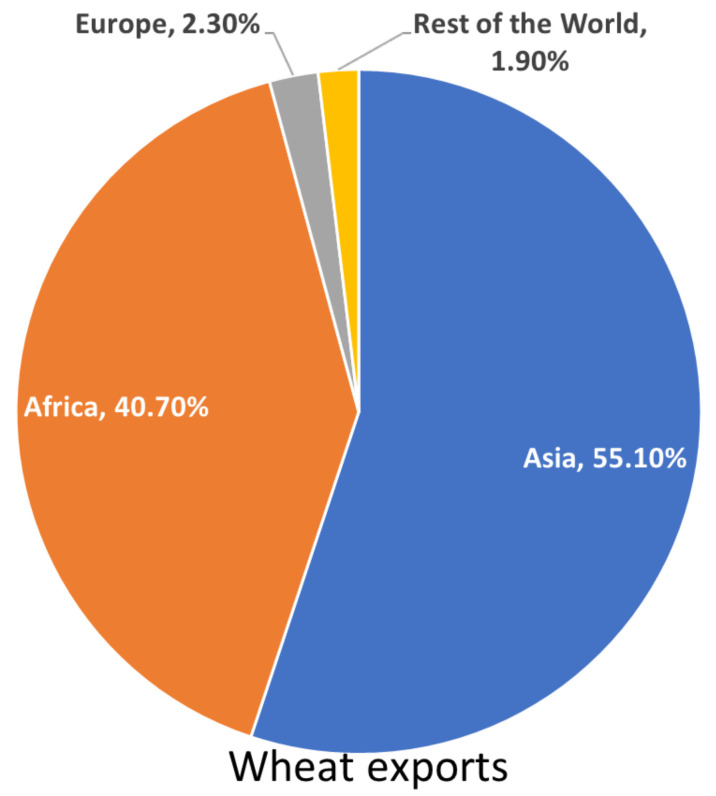
Ukrainian wheat exports by destination in 2021 [52].

The role of Eastern Europe and Central Asia as a potential new “breadbasket” for the world has been called into question due to previous export restrictions affecting the reliability of Russia, Kazakhstan, and Ukraine (RUK) as exporters [49]. Ukrainian wheat would be harvested primarily in July and August, and last year the 16 MMT of exports were relied upon heavily by lower-income countries, with Morocco, Bangladesh, Pakistan, Egypt, and Indonesia each importing over a million tonnes of Ukrainian wheat from the 2021 harvest [50]. Over 95% of Ukrainian wheat exports were to be sent to countries in Africa or Asia. This wheat was to be primarily used in direct consumption and was expected to be a significant source of calories during the summer of 2022 in Northern Africa and South Asia [51] as shown in Figure 7. Countries such as Egypt have already intervened in the market, but absolute shortages remain a possibility [52].

Ukraine was the world’s largest exporter of sunflower oil in 2021. This value is significant, having grown to over $6 billion in 2021. Despite the fact that the war threatens to remove billions of dollars and trillions of calories from the global marketplace through the disruption of Ukrainian seed oil shipments, the impact on the consumer is likely to be attenuated. Edible fats remain extremely fungible, with other oils substituting for a sunflower oil well. Oils can also be stored for long periods of time, and major blocs such as the EU and the United States maintain significant reserves.

Pharmaceutical production in Ukraine had enjoyed double-digit growth for years prior to the war, but Ukraine remained heavily dependent on foreign sources for needed medications [53]. As of 2019, packaged medications accounted for $1.8 billion in imports, exceeding exports by more than ten to one and comprising a super majority of medications consumed in the country. Between Ukrainian refugees and internally displaced Ukrainians, there are already over ten million Ukrainians who will not be able to get medications through their previously normal channels, and that number is liable to increase. Pharmaceuticals are significantly less fungible than food or other commodities as regulatory environments differ spectacularly across national borders. The State Service of Ukraine on Medicines and Drug Control is not part of the European Medicines Agency, and differences exist in the required labeling, dosage, and formulary of medication for distribution in Ukraine versus neighboring countries in the EU. At least $2 billion worth of pharmaceuticals will probably need to find new supply chains in 2022 because of the conflict.

Temporary shortages of specific medicines are likely but almost certain to be minor in the face of a $1.2 trillion global pharmaceuticals market. Of greater concern are the tens of millions of people within Ukraine who will have uncertain access to supply chains for needed medicines for the duration of the war. This will lead to considerable excess death as much of the Ukrainian population is over forty years old.

Nowhere is the disconnect between the importance of a supply chain and its financial cost more dramatic than it is with potable water. Pipelines carrying potable water for millions of Ukrainians had already been threatened and disrupted by Russian military and paramilitary actions for years before the 2022 invasion [54], but in March 2022 the United Nations noted that wartime activities had left at least 5% of the people of the Donetska Oblast without access to water [55]. Further, the water resources of neighboring Poland were already under moderate stress [56]. While water is not financially expensive, local sources sufficient to replace the water sources for tens of millions of Ukrainians simply do not exist. Deaths related to compromised water supplies could easily reach hundreds of thousands, despite the low cost of restoring stable water access once eventually achieved.

Ukraine produced meat primarily for internal consumption, predominantly poultry and pork. While Ukraine was a net exporter of meat, roughly 2.2 MMT of meat was produced for consumption within Ukraine on a yearly basis. The disruption of Ukrainian animal agriculture represents a substantial impact on food security for the Ukrainian people, although its impact on world poultry prices will be minimal. The impact of the direct loss of Ukrainian meat imports will be minor compared to the indirect effects of the global rising cost of animal feed due to the disruption in Ukrainian maize [57].

All in all, the Ukrainian people should consume over 80 billion calories every day to maintain the health of the people. Trollman et al. [58] confirmed that food insecurity during the COVID-19 pandemic had minimal effect on consumer food preferences or willingness to seek nutritionally adequate substitutes or alternative foods. Similarly, price volatility of food coupled with decreasing purchasing power is likely to make balanced nutrition and healthy diets a problem in Ukraine [59], especially when combined with known tendencies of non-rational Ukrainian consumer behavior [60]. Psychological distress has been found to be associated with less frequent consumption of meat, fish, vegetables, fruit, and animal fat in post-Soviet republics [61]. For each person killed directly in the war, nine may be killed indirectly; with child and birth-related mortality significantly affected and infectious diseases at risk of re-emerging [62].

As Ukraine has historically been a net food exporting country, almost none of the supply chain infrastructure to provide that from external sources exists. The loss of Ukrainian contributions to the global food marketplace is felt the world over, but the looming food insecurity of the Ukrainian people is an often-overlooked problem. The displaced people of Ukraine number more than the entire populations of neighboring Belarus, Slovakia, Hungary, or Moldova, and there are no established means for them to acquire needed food, water, or medications. Such supply chains will need to be built from scratch in the face of an ongoing Russian invasion.

#### 3.2.5. Impact on Food Dependent Services

The Russian invasion of Ukraine has been associated with the fastest displacement of people since the Second World War, with over ten million people internally displaced or international refugees in just one month of conflict [63]. Displaced people have historically had lower labor productivity and higher rates of informal employment than non-displaced people or voluntary migrants [64]. These disadvantages are generally reduced over time, but there is reason to expect a forced migration of this speed and magnitude will present a more difficult problem of assimilation at destination than a smaller or slower displacement of people.

Historical examples are uncommon, but there are precedents set by the 1971 Bangladesh Genocide [65] and the Korean War of 1950 [66]. The per capita GDP of Bangladesh caught up to and exceeded that of Pakistan in 2016, some forty-five years after the displacement of over thirty million people during the conflict [67]. The Korean War improved on that performance, although even then the displacement of people on the Korean peninsula began in 1950 and the Republic of Korea’s legendary economic performance began after democratization in 1987. Notably, North Korea was never democratized and remains economically impoverished 72 years later. Examples of comparable mass forced displacement with economic recovery taking substantially less than two generations are available from World War II, but all of those examples involve very large investments from the United States or the Soviet Union. Without strong external intervention to rebuild, Ukraine will likely remain impoverished by this conflict for generations to come.

A plausible scenario is one where some portion of Ukraine remains under Russian occupation for a protracted period of time. The economic prospects of those occupied regions are fairly bleak. The Russian Federation is already maintaining multiple occupied frozen conflict zones, creating obvious comparison points. Both the Transnistria region in Moldova [68] and the South Ossetia region in Georgia [69] boast a per capita GDP that is less than half that of the unoccupied portions of their respective countries.

Labor productivity is hampered in multiple ways. Forced migration removes workers from employment appropriate to their skills, and it is often difficult for them to find equivalent employment at their destination. Employment vacancies that do exist become more difficult to fill when potentially qualified candidates are evacuating. The war is also destroying infrastructure at a tremendous rate, with estimates ranging from $63 billion [70] to nearly ten times that [71]. Far beyond the direct financial losses, the destruction of physical and human capital will severely impact labor productivity immediately and hobble growth for the foreseeable future.

**Figure 8:**
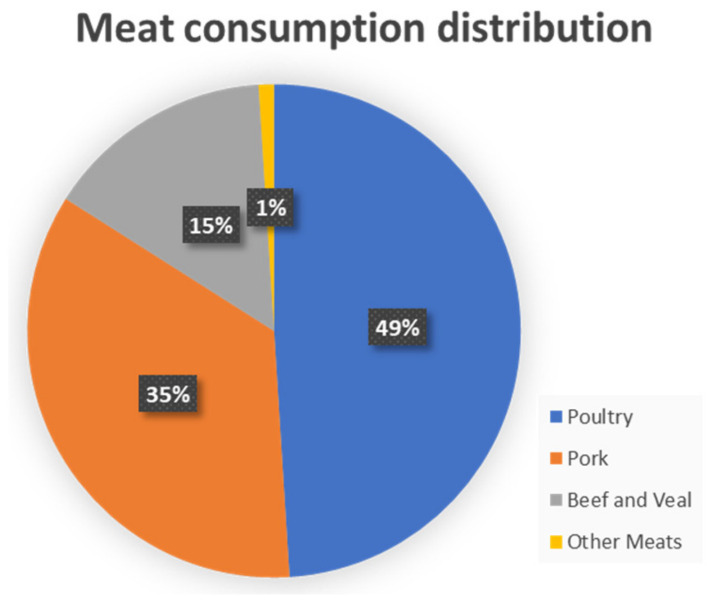
Meat consumption in Ukraine in 2020. The per capita figures are 26 kg for poultry, 19 kg for pork, 8 kg for beef and veal, and 1 kg for other meats [73].

Not only humans, but also livestock face mass displacement in times of war. The Ukrainian agricultural sector primarily produces meat for internal consumption, and the Ukrainian people consumed poultry, pork, and beef for 99% of their meat consumption (Figure 8). Almost all of the chicken, pigs, and cattle of Ukraine are likely to be slaughtered within the next two years. Some of this meat will reach consumers and some will go to waste. The substantial majority of these animals are industrial breeds functionally indistinguishable from those in use in Poland or Hungary, meaning that farms and herds will be quickly repopulated as soon as the country is again able to consistently supply animal feed. However, there are Ukrainian legacy breeds such as the Ukrainian Grey Cattle that are already endangered with few examples outside of Ukraine [72]. These breeds may go extinct entirely, and future generations would see them only in historical texts.

Almost 90% of Ukraine’s poultry are industrial broilers. Worldwide there are over 23 billion such birds, and a typical bird spends 21 days as an egg and just 42 days out of the egg before slaughter [74]. The cycle can easily be discontinued in the face of a projected lack of feed, and additional eggs can be incubated when feed is available. The poultry industry is very agile and can adapt to changes in feed maize supply much more quickly than the maize farmers can. Poultry production can be up to normal levels within months of a successful maize harvest. Beef cattle grow much more slowly, with calves growing to adulthood over months and years [75]. The expected 2022 mass slaughter of beef cattle will impact Ukrainian cattle farming for years. Cattle farming would likely not recover until three or four years after access to consistent animal feed was reestablished. 

In the intervening years between the eventual return of peace and stability to Ukraine and the reestablishment of a domestic beef industry, the capacity for feeding and caring for full-grown and near full-grown cattle will exist in Ukraine. This capacity can be used for finishing and fattening animals raised in other countries. An independent Ukraine may be an attractive option for farmers in currency-limited and sanction-restricted countries, as beef cattle finished on Ukrainian grain will be considered Ukrainian and become eligible to be sold on to European consumers, regardless of the sanctions imposed on the country cattle were calved in.

Indirect losses to productivity in other countries are also a certainty. Ukraine has historically been one of the major sources of fertilizers in Europe, accounting for as much as 25% of all fertilizers produced on the continent in 2010 [76]. While the last decade has seen a significant reduction in Ukrainian fertilizer exports, Ukraine continued to export hundreds of millions of dollars of fertilizers per year prior to the war. The loss of this production threatens more than the financial value of the fertilizers, but potentially the productivity of agriculture in other parts of the world [77].

Neighboring Belarus exports over $2 billion worth of potassium rich fertilizers every year. While there is currently no reason to believe that Russian or Ukrainian troops will destroy Belarusian potash production, there may be sanctions that hinder or delay the export of fertilizers from Belarus. Most of the Belarusian fertilizer is intended to be exported to Brazil, China, India, or Indonesia, and none of those countries are currently preventing trade in goods from Belarus. However, the sanctions remain fluid, and Belarusian goods may be partially or wholly blockaded by third countries whether the would-be recipient countries are signatories to sanction regimens or not.

#### 3.2.6. Impact on Food Quality

Countries worldwide are barely recovering from the disastrous economic consequences of the global COVID-19 pandemic, and the ongoing Russian invasion of Ukraine could make this economic situation even worse, particularly in the global food supply chain. These economic impacts on the food supply chain are predicted to have a substantial influence on food safety and quality in various ways, including price increases of energy causing rising prices of food, feed, and fertilizers, and changes in seasonal worker availability, among others [78]. Although studies have focused on the effect of the invasion of Ukraine by Russia on the food supply chain from an economic point of view [79], very little academic research has been done on the impact of this crisis on food quality and safety.

Historically, wars and other major violence tended to have both direct ruinous impacts on food security and indirect severe consequences on food quality and safety, with lasting effects over time. For example, a recent study discussed the impact of the Syrian conflict on crop production and food security in Syria, even more than ten years after the start of this conflict [80]. Another study described the catastrophic consequences of the war in Yemen on food security and nutrition status, after several years of conflict in this country [81]. The Russian invasion of Ukraine is unlikely to be an exception.

As Ukraine has one of the biggest nuclear industries in the world [82], the question of maintaining its nuclear facilities has become of urgent importance, only a few days after the Russian invasion of this country. Especially the shelling of the Zaporizhzhia power station, the largest in Europe, by Russian armed forces has triggered unprecedented global outrage, as nuclear experts have warned that Ukrainian nuclear sites are at risk of a nuclear disaster due to the release of some radioactive material [82,83]. Indeed, there has been a fear of a similar catastrophe to the one that happened in 1986 at Chernobyl, the site of the world’s worst nuclear disaster, with long-term devastating effects on living plants, animals, and the environment. Recently, it has been shown that, even 35 years after the Chernobyl disaster, contamination levels in many foods still exceed acceptable limits [84]. The Chernobyl nuclear power plant has again become a widespread threat after Russian shelling at the site, but a recent report from the International Atomic Energy Agency confirmed that the radiation levels near the Chernobyl plant are within safe limits [85,86].

Another food safety-related issue that could be directed affected by wars is the spread of diseases. FAO highlighted the significant risk of proliferation of animal diseases, such as African swine fever (ASF) in Ukraine and neighboring countries [87]. The increasing risk of ASF was explained by the negative impact of the conflict on the existing capacities in the areas of surveillance, diagnostics, vaccination, inspection services, veterinary care facilities, pig farm management, among others. In addition, the spread of ASF can be enhanced by the massive movement of refugees and displaced people fleeing from the conflict. Moreover, the Russian invasion of Ukraine is expected to put more pressure on fertilizers’ availability and promote the use of alternatives such as animal manure. This could lead to less crop yield and reduced quality due to less protection against fungal and bacterial infections [87,88,89].

The Russian invasion of Ukraine and sanctions against Russia and Belarus will increase the global demand for food and increase food prices, which may impact food quality and safety. Price spikes caused by the reduction of food availability are projected to be accompanied by impaired product quality due to a possible shift in consumption patterns towards cheaper food products but often with lower quality. For example, the declines in Russian exports will require food companies to omit or substitute certain ingredients or change the manufacturing process or recipe and find alternatives in the short term, which may lower the quality of the products, as these alternatives in some cases may not meet desired product specifications (e.g., GMO-free) or EU legislations [89,90]. Besides, as extreme events often spark waves of “panic buying” such as the one that emerged with the outbreak of COVID-19 during the lockdown periods [91,92,93], similar shopping behavior can be expected with conflicts and wars. Purchasing extra food and an increase in food stocks at home (panic stockpiling) could lead to more food waste. 

Food fraud and food adulteration issues have at times been reported in countries with conflicts. The key characteristics of food fraud are non-compliance with food laws, and misleading the consumer for financial gain. Food fraud can include a substitution of species, adding or replacing food ingredients with undeclared substitutes, and discrepancies in food production or farming method [94,95]. Occurrences of food fraud are likely to become more prevalent during the COVID-19 pandemic [96]. Similarly, the Russian invasion of Ukraine could increase the opportunities for food fraud and may impact global food traceability systems. Honey, sunflower oil, grains, and seafood are among the main foodstuffs produced in Ukraine and that are most vulnerable to food fraud.

Ukraine is the largest producer of honey in Europe, with more than half of Ukrainian honey being exported [97,98]. Honey is a product that is subjected to frequent adulteration in several ways, such as mislabeling, falsely declaring the botanical or geographical origins, direct or indirect addition of cheaper sweeteners or low-quality honey [99]. Due to adulterated imported honey that continues to flood the market, domestic honey producers find difficulties in building a sustainable business model predicated on authentic honey [100]. Ukraine and Russia are leading exporters of sunflower oil. The invasion of Ukraine provides a unique opportunity for fraudsters to replace sunflower oil with refined rapeseed oil, mineral oil, castor oil, cottonseed oil, among others [98,101]. The seafood supply chain is particularly vulnerable to fraud, such as substituting expensive varieties of fish with low-value species, mislabeling the geographical origin, labeling cheaply farmed fish as more expensive wild-caught fish, or labeling traditionally farm-raised fish as more valuable ecological or organic varieties [95,100]. As seafood (including whitefish, such as Atlantic cod and haddock Seafood) is among Russia’s biggest agricultural exports, restrictions on Russia’s exports of these products are likely to impact supply and increase fraud. Some grains, especially wheat and maize, as well as pulses are also expected to be highly affected by this conflict, as can be seen in Table 4.

## 4. Discussion

Food supply has too often been a politically driven resource restricted by conflict and war. The quick solution to this crisis is to halt the war and allow agricultural production to resume immediately and safely to avoid further potential impacts on food security in Ukraine—and beyond—in the coming days, weeks, and months.

Food production, processing, and storage have been shown by this research to be most under pressure from disruption to planting and harvesting which includes the availability of agricultural labor and agricultural inputs. The identified solution given continued conflict is to minimize disruption to global supply chains by finding alternative sources for the lost crops. This aligns with the suggestions of the FAO [13] for undertaking a number of actions to alleviate the food security challenge, firstly focusing on resuming agricultural production and protecting farmers. Particularly, the spring sowing campaign for maize, barley, and other crops, as well as the summer harvesting of winter crops, is of paramount importance. Bentley [18] recommends expanding wheat production in high-productivity areas (North America and Europe) and in regions with suitable conditions (Sudan and Nigeria are promising), and by increasing productivity in places where it is low (such as Ethiopia and Turkey). Also, more efforts are needed to promote novel food production systems such as aquaponics, aeroponics, and hydroponics nearer to home, although the benefits may only be realized in the long term.

This research also indicates that food-dependent services requiring human labor and/or livestock are likely to be significantly affected. Increasing support for innovations and digital transformation would alleviate human labor issues. However, the human-livestock interrelated issues are difficult to solve while conflict continues, and culling is a likely outcome. If possible, livestock needs to be protected and have access to animal feed, fodder, and veterinary medicine to avoid further stock reduction Following food production, impacts on food transport logistics are suggested to be alleviated by initiating alternative sources of supply and bringing supply chains home to ease over-reliance on risky foreign suppliers. Improving the flexibility of transport logistics with respect to global food supply chains would also be beneficial. Establishing a policy for maintaining a ‘safety stock’ of critical food commodities should be undertaken at a national level.

For effects on the food market/retail, and consumers, it will be essential to know the level of inflation on food products and consumer costs to build strategies to preserve profitability without compromising affordability.

This research has also indicated that food quality may suffer as a result of the conflict. The need for cybersecurity has risen due to the state-sponsored cyber-attacks and the increasing level of automation in the supply chain. Devising a plan for workforce safety and bracing for disruption to logistics and supply chain partners would be key. Therefore, food supply chain stakeholders should work together to determine and decide on the medium to a long-term course of action.

## 5. Conclusions

This article analyzed the impact of the Russia-Ukraine conflict on the global food supply chains. The hypothesis underlying this research has been confirmed in that there will be significant impacts on the effectiveness and responsiveness of global food supply chains as a result of the conflict between Russia and Ukraine. Six key areas of the food supply chain that would be impacted due to the ongoing war were highlighted: (a) Food production, processing, and storage, (b) Food transport logistics, (c) Food market/retail, (d) Consumers, (e) Food dependent services, and (f) Food quality. Research showed that although this conflict will affect the majority of the economies, however, the most affected economies are in Europe and Africa. It is advisable for these adversely affected economies to explore and find alternative food supply chain partners and solutions in North America, South America, the Middle East, Australasia, and some regions of Asia and Africa that have been less affected by this conflict. This article has also briefly suggested solutions and strategies for the global food supply chains to counter similar warlike situations.

## Figures and Tables

**Table 1:** Inclusion and exclusions criteria for the literature review.

Inclusion Criteria	Exclusion Criteria
Available in English	Unavailable in English
Published by a government, NGO, or broadsheet	Published by a tabloid
Published by an expert in the field	Published as a generic blog
Related to identified impact categories	Unrelated to identified impact categories

**Table 2:** Major agricultural products in Ukraine affected by the war. Based on data from [13].

Agricultural Product	Seasonality	Major Concerns and Impacts
Grains	Winter grain: planting from September to October, harvesting from July to August	Half of the winter wheat and 38% of rye to be harvested in summer 2022 are in occupied or war-affected areas
Oilseeds	Sunflowers: planting in April, harvesting from mid-September to mid-October	Sunflowers planting will be seriously affected in at least nine Oblasts (regions). Sown under sunflowers in 2022 may be 35% lower compared to 2021
Vegetables	Land preparation for vegetables from late February to March, sowing from mid-March to mid-May, harvesting from July to mid-September	Disruptions due to lack of inputs, lack of access to land and concerns over safety for workers will affect summer harvest
Livestock	Seasonal with respect to availability of feeds and forage, and breeding cycles with respect to eggs, dairy, and meat	Disruptions due to lack of feed, feed additives, veterinary medicine, and breeding stock; insecurity of livestock transportation; as well as damage to infrastructure; will affect the livestock sector for as long as the war continues

**Table 3:** Importation of Ukrainian and European wheat, maize, sunflower seed and oil in 2020.

Country	Imported Wheat (tonnes)			Maize (tonnes)			Sunflower Seed (tonnes)			Sunflower Oil (tonnes)		
	Total	EU	Ukraine	Total	EU	Ukraine	Total	EU	Ukraine	Total	EU	Ukraine
Austria	1,151,669	1,151,302 (99.97%) ^1^	286 (0.02%) ^1^	967,258	887,402 (91.74%)	30 (0.003%)	156,041	150,733 (96.60%)	29 (0.02%)	76,115	59,975 (78.80%)	6579 (8.64%)
Belgium	-	-	-	1,922,955	1,502,583 (78.14%)	418,921 (21.79%)	119,686	112,493 (93.99%)	2536 (2.12%)	586,818	574,233 (97.86%)	10,738 (1.83%)
Bulgaria	-	-	-	27,844	20,454 (73.46%)	209 (0.75%)	1,020,755	457,030 (44.77%)	182,539 (17.88%)	41,875	12,635 (30.17%)	25,992 (62.07%)
Croatia	-	-	-	-	-	-	-	-	-	69,080	23,905 (34.60%)	104 (0.15%)
Cyprus	-	-	-	289,032	188,490 (65.21%)	29,570 (10.23%)	-	-	-	7234	4663 (64.46%)	2517 (34.79%)
Czech	-	-	-	-	-	-	210,192	198,102 (94.25%)	2789 (1.33%)	58,985	50,830 (86.17%)	6690 (11.34%)
Denmark	-	-	-	-	-	-	16,348	15,369 (94.01%)	211 (1.29%)	17,944	14,997 (83.58%)	104 (0.58%)
Estonia	-	-	-	25,984	23,985 (92.31%)	777 (2.99%)	6066	872 (14.38%)	4515 (74.43%)	7036	1378 (19.58%)	2355 (33.47%)
France	-	-	-	661,440	641,541 (96.99%)	6 (0.001%)	325,638	234,358 (71.97%)	501 (0.15%)	298,341	126,944 (42.55%)	154,758 (51.87%)
Germany	3,999,369	3,978,627 (99.48%)	5066 (0.13%)	3,802,900	3,378,093 (88.83%)	393,290 (10.34%)	389,117	363,570 (93.43%)	2478 (0.64%)	495,001	477,005 (96.36%)	14,854 (3.00%)
Greece	894,928	625,470 (69.89%)	86,892 (9.71%)	-	-	-	-	-	-	69,435	53,988 (77.75%)	14,388 (20.72%)
Hungary	-	-	-	168,688	110,533 (65.53%)	25,675 (15.22%)	131,494	108,742 (82.70%)	7 (0.01%)	46,795	14,170 (30.28%)	21,874 (46.745)
Ireland	-	-	-	1,313,414	118,007 (8.98%)	415,402 (31.63%)	-	-	-	-	-	-
Italy	7,994,393	4,894,209 (61.22%)	233,869 (2.93%)	5,994,600	4,586,124 (76.50%)	770,245 (12.85%)	159,738	139,130 (87.10%)	2667 (1.67%)	589,558	185,720 (31.50%)	346,749 (58.82%)
Latvia	-	-	-	93,839	17,912 (19.09%)	5462 (5.82%)	5988	1822 (30.43%)	208 (3.47%)	14,014	7649 (54.58%)	3194 (22.79%)
Lithuania	-	-	-	321,350	10,321 (3.21%)	217,387 (67.65%)	7626	3989 (52.31%)	728 (9.55%)	36,950	4185 (11.33%)	17,155 (46.43%)
Malta	-	-	-	-	-	-	-	-	-	2573	1063 (41.31%)	1408 (54.72%)
Netherlands	4,296,917	4,189,485 (97.50%)	30,920 (0.72%)	5,945,756	2,748,433 (46.23%)	3,027,455 (50.92%)	768,103	738,468 (96.14%)	1421 (0.19%)	885,606	202,459 (22.86%)	679,591 (76.74%)
Poland	869,332	860,548 (98.99%)	3946 (0.45%)	421,653	379,173 (89.93%)	1500 (0.36%)	65,622	49,675 (75.70%)	5555 (8.47%)	219,866	77,360 (35.19%)	140,210 (63.77%)
Portugal	-	-	-	1,899,504	370,484 (19.50%)	732,523 (38.56%)	-	-	-	67,220	39,710 (59.07%)	14,929 (22.21%)
Romania	-	-	-	1,348,879	1,238,860 (91.86%)	689 (0.05%)	242,832	59,545 (24.52%)	1877 (0.77%)	54,517	27,425 (50.31%)	12,645 (23.19%)
Slovakia	-	-	-	88,878	85,213 (95.88%)	3549 (3.99%)	-	-	-	43,068	38,045 (88.34%)	3929 (9.12%)
Slovenia	-	-	-	776,976	191,535 (24.65%)	1598 (0.21%)	-	-	-	-	-	-
Spain	4,151,812	3,436,105 (82.76%)	373,294 (8.99%)	8,067,136	2,512,059 (31.14%)	2,719,175 (33.71%)	402,353	331,560 (82.41%)	983 (0.24%)	604,241	125,497 (20.77%)	430,633 (71.27%)
Sweden	-	-	-	-	-	-	-	-	-	38,728	33,502 (86.51%)	1795 (4.63%)

^1^ % of total imported. Data adapted from FAOSTAT [21].

**Table 4:** Impact and risk associated with certain food products that are particularly affected by the Russian invasion of Ukraine.

Products	Global Export: Combined Russia/Ukraine	Short Term Impact	Long Term Impact	Risks	Reference
Wheat	30%	Reasonable supplies	Serious shortages	Adulteration with high protein flour, allergens, lower quality grains, and mycotoxins	[98]
Maize	20%	Shortages	Uncertain	Lower quality grains and mycotoxins	[98]
Pulses	Among the top 5 producers	Reduced supplies	Shortages	Adulteration, allergens	[98]
Sunflower Oil	80%	Pressure on alternative vegetable oil sources	Uncertain	Adulteration with other less expensive oils such as palm oil, mineral oil, and rapeseed oil	[98,101]
Honey	Number one producer in Europe	Pressuring supply and demand	Contamination from conflict chemicals (e.g., heavy metals/biological hazards)	Adulteration with many cheaper substances (such as sugar syrups or other alternatives) and health risks due to contaminants	[98,99]
Fish	40% whitefish (e.g., 30% Atlantic cod and 25% haddock)	Price increases and reduced supply	Impact on seafood processing industries	Substitution of species, incorrect origin, etc. traceability)	[98,100]
Fertilizer	The largest exporter of urea and potash	Price increases	Reduce crop yields	Fertilizer adulteration	[87,98]

## Data Availability

Not applicable.
